# The Role of Hedgehog Pathway in Female Cancers

**DOI:** 10.26502/jcsct.5079089

**Published:** 2020-10-09

**Authors:** Natalia Garcia, Mara Ulin, Ayman Al-Hendy, Qiwei Yang

**Affiliations:** Department of Surgery, University of Illinois at Chicago, Chicago, Illinois, USA

**Keywords:** Hedgehog pathway, Female cancer, Ovarian cancer, Cervical cancer, Endometrial cancer, Uterine leiomyosarcoma, Therapeutic option

## Abstract

**Background:**

The hedgehog pathway (HH) is one of the key regulators involved in many biological events. Malfunction of this pathway is associated with a variety of diseases including several types of cancers.

**Methods:**

We collected data from public databases and conducted a comprehensive search linking the HH pathway with female cancers. In addition, we overviewed clinical trials of targeting HH pathway in female cancers.

**Results:**

The activation of HH pathway and its role in female cancers, including breast cancer, ovarian cancer, cervical cancer, endometrial cancer, and uterine leiomyosarcoma were summarized. Treatment options targeting SMO and GLI in HH pathway were reviewed and discussed.

**Conclusions:**

The hedgehog pathway was shown to be activated in several types of female cancers. Therefore, targeting HH pathway may be considered as a therapeutic option to be acknowledged in the treatment of female cancers.

## 1. Introduction

The hedgehog (HH) gene was described by Christiane Nusslein-Volhard and Eric Wieschausin in 1980 during gene screening in *Drosophila* [1]. The HH signaling pathway plays an essential role in embryonic and normal tissue development along with patterning and tissue differentiation [2]. The role of dysregulated Hedgehog Signaling pathway in cancer was first identified in basal cell nevus syndrome, a genetic disorder characterized by an increased risk of basal cell carcinoma and medulloblastoma. From these findings, it is understandable that mutations in PTCH1 caused aberrant activation of HH pathway and further predisposed patients to develop cancer [3].

## 2. Hedgehog Pathway

**Figure 1:**
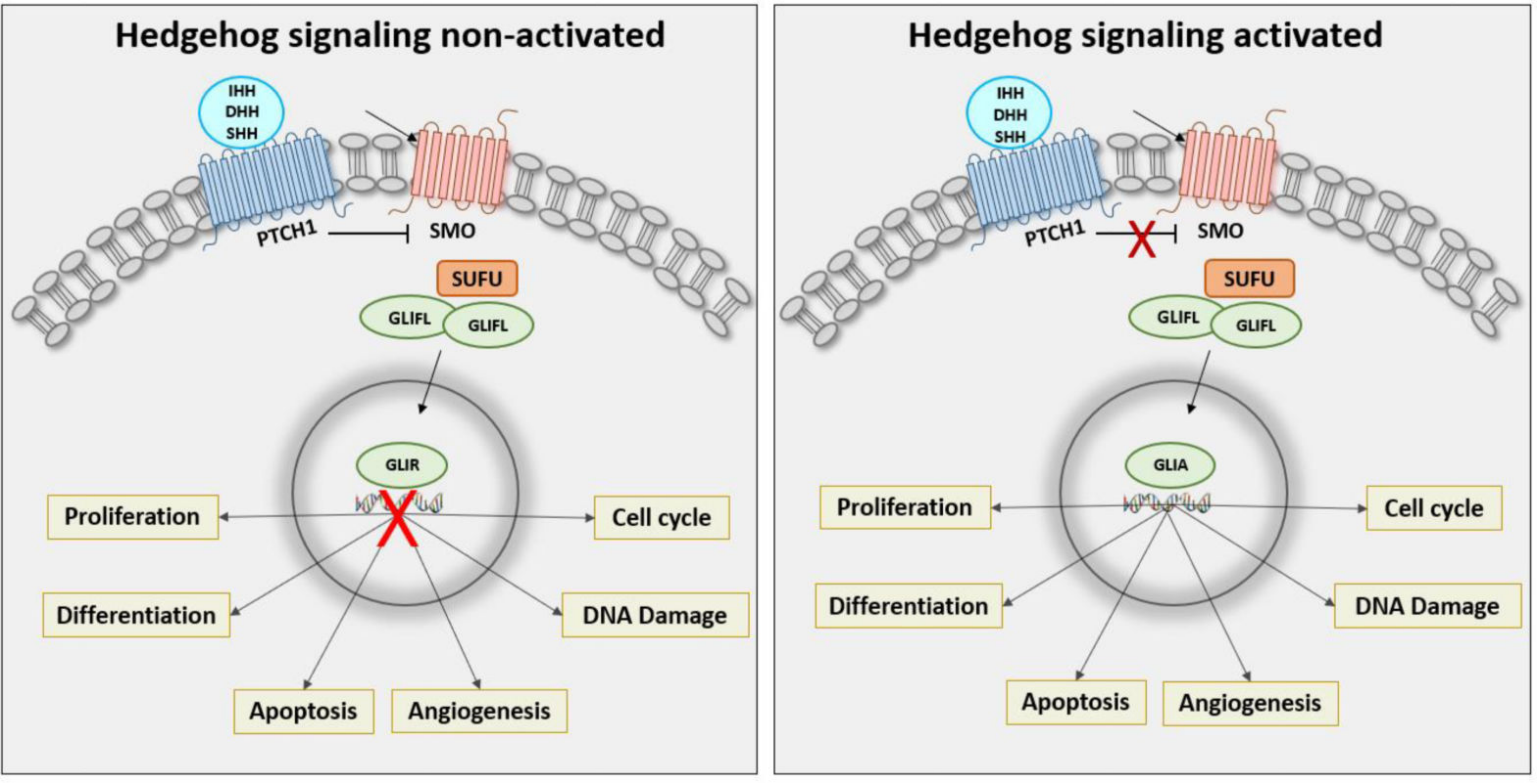
Non-activated or Activated State of Sonic Hedgehog Signaling Pathway. **Left panel:** With the absence of HH ligand, PTCH1 inhibits SMO activity. GLI activity is phosphorylated, converting GLI full- length (GLIFL) to repressor form (GLIR). GLIR translocates into the nucleus, which binds to HH target gene promoters and suppresses their expression. **Right panel:** The activation of the signaling occurs when HH ligands bind to PTCH, HH relieves the inhibition of PTCH to activate the signal transduction. SMO transmits a signal to the cytoplasm in a phosphorylation cascade leading translocation of GLI activator (GLIA) to the nucleus and binding to the target gene promoters and activates the transcription of the target genes.

In vertebrates, HH signaling pathway consists of 3 ligands which are Indian hedgehog (IHH), Desert hedgehog (DHH) and Sonic hedgehog (SHH), a receptor called Patched 1 (PTCH1), a signal transducer called Smoothened (SMO), a cytoplasmic protein named SUFU and 3 transcription factor (GLI1, GLI2 and GLI3) [4] (Figure 1). Alteration in HH signaling pathway promotes GLI translocation into the nucleus leading to overactivation of several target genes which regulate cell differentiation (*INSM1, SOX2, OCT4 and NANOG*) [5–7], proliferation (*c-MYC* and *n-MYC*) [8, 9], apoptosis (*BCL2, CASPASE 3, BAX, CASPASE 9* and BAK) [8–12], cell cycle (*CCND1* and *P21*) [8, 11, 13–15], DNA damage (*RAD51* and *TP53*) [15, 16], angiogenesis (*c-MET, VEGFR2*) [11, 17] and adhesion (*N-CADHERIN, E-CADHERIN* and *SNAIL 1*) [13, 18, 19] contributing to the pathogenesis of cancer. The hedgehog signaling pathway is tightly associated with embryogenesis as well as with the development of several female cancers. This review summarizes the findings of the role of HH signaling in female cancers and outlines the treatment options.

## 3. Hedgehog in Female Cancers

**Figure 2:**
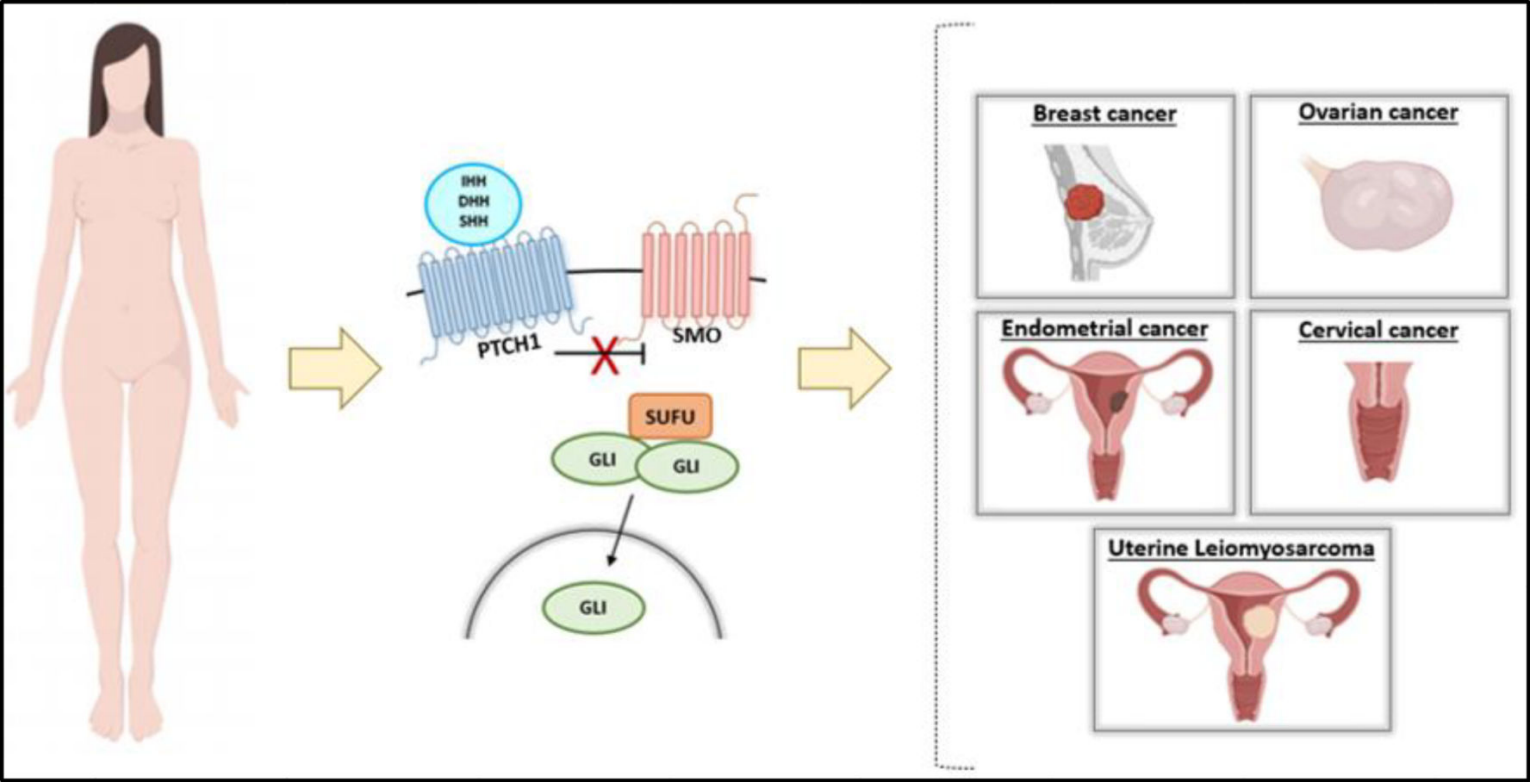
Abnormal Hedgehog Signaling Pathway Leads to Female Cancers.

HH signaling pathway has been reported to be involved in the pathogenesis of several types of female cancer, including breast [20], ovarian [21], endometrium [22], cervical [23], and uterine leiomyosarcoma [24] (Figure 2).

### 3.1 Breast cancer

Breast cancer is the most frequently diagnosed cancer in women and the second leading cause of death in women diagnosed with cancer [25]. The HH signaling pathway plays an essential role in mammary gland development. Throughout a lifetime, this pathway activity varies. For instance, in the early phases of embryogenesis, this pathway is repressed to allow proper mammary gland parenchyma formation. During puberty there is ductal morphogeneisis and the HH signaling pathway is required for activation to promote the elongation of the terminal buds. Soon after puberty, in the mammary glands, the HH signaling pathway activity decreases [26]. In breast cancer HH signaling pathway activation has been associated with younger age presentation (<50 years), larger tumor size, lymph node metastasis, progesterone receptor-negative status, high proliferation index of Ki67, and poor overall survival [27–29]. Studies have shown that the expression of GLI1 [29–31] along with GLI1, 2 and 3 protein levels are upregulated in breast tumor compared to normal tissue [28]. Furthermore, the GLI expression level is associated with a higher tumor grade [29]. It is reported that targeting HH pathway in breast cancer showed promising results in several clinical trials (Table 1).

### 3.2 Ovarian Cancer

Ovarian cancer is the leading cause of death from gynecologic malignancies in the United States [32]. Epithelial ovarian cancer accounts for over 90% of all ovarian malignancies and comprises five histological subtypes: serous, mucinous, endometrioid, undifferentiated and clear cell type [33]. Aberrant activation of the HH signaling pathway is mediated through increased endogenous ligand-dependent expression of HH or ligand-independent mutations of PTCH, SMO and SUFU [34, 35]. Accumulating evidence suggests that the deregulation of the HH signaling pathway also contributes to the malignancy of ovarian cancer [36–40]. The expression of SHH, DHH, GLI, PTCH and SMO is absent in normal ovary [38, 41]. Elevated expression of PTCH1 and GLI1 is correlated with poor prognosis in ovarian cancer [41, 42]. In addition, the presence of SHH, DHH, PTCH, SMO and GLI1 proteins are associated with abnormal cell proliferation [38]. Moreover, the HH pathway is involved in regulating cancer stem cells leading to tumor formation, progression and invasion in ovarian cancer [43, 44]. It is well demonstrated that the strategy for blocking this pathway has been used in several clinical trials (Table 1) with a promising outcome.

### 3.3 Cervical cancer

Cervical cancer is the third most common malignant neoplasm in females, representing the fourth cause of cancer deaths among females worldwide [45]. Persistent infection by high-risk HPVs (16,18,31 and 33) is a risk factor for the development of cervical cancer [23, 46–48]. High expression of the HH signaling pathway regulates proliferation, migration and invasion of cervical cancer cell lines [23]. Reports in the literature have demonstrated that inhibition of the HH signaling pathway with Cyclopamine and Gant 58 decreased invasion and enhanced apoptosis, demonstrating this treatment can be effective in treating cervical cancer [49].

### 3.4 Endometrial cancer

Endometrial cancer is the most common malignancy of the female reproductive tract, with a substantial increase in incidence and mortality rate in developed countries. This type of cancer predominantly affects postmenopausal women. However, 15–25% of cases are diagnosed before menopause. Many risk factors have been identified to predispose women with endometrial cancer, including polycystic ovarian syndrome, obesity and endometrial hyperplasia [50]. Moreover, several pathways have been identified to be altered in endometrial carcinoma including HH signaling pathway. Interestingly, PTCH1 has been found to be expressed in patients with endometrial hyperplasia, and GLI 1, GLI2, cytoplasmic GLI3 and SUFU have also been identified to be overexpressed in patients with endometrial carcinoma [51–53].

### 3.5 Uterine leiomyosarcoma

Uterine Leiomyosarcoma (LMS) is the most common type of uterine sarcoma. This tumor can be present at any age. However, it is frequently diagnosed in the perimenopausal years. It represents around 3–7% of all uterine cancers [54]. LMS is an extremely aggressive tumor that shows a challenge for treatment. LMS exhibits resistance to standard therapy [55]. The involvement of the HH signaling pathway in uterine leiomyosarcoma was first described in 2016 [24]. Elevated expression of SMO and GLI 1 was observed in leiomyosarcoma when compared to normal myometrium and uterine fibroids tissue. In addition, SUFU and SHH proteins were correlated with poor prognosis in leiomyosarcoma patients [24]. Recently, we demonstrated that uterine leiomyosarcoma cells exhibited an upregulation of SMO and GLI1 members concomitantly with an increase in nuclear translocation of GLI-1 and 2 compared to uterine smooth muscle cells. Uterine cells showed a decrease in proliferation, migration, invasion and exhibited an increase in apoptosis in response to treatment with SMO and GLI inhibitors, respectively [56, 57]. Identifying the HH pathway in relation to this aggressive cancer might allow better treatment options for women suffering from this devastating condition.

## 4. Therapeutic Options to Block the Hedgehog Pathway

**Figure 3:**
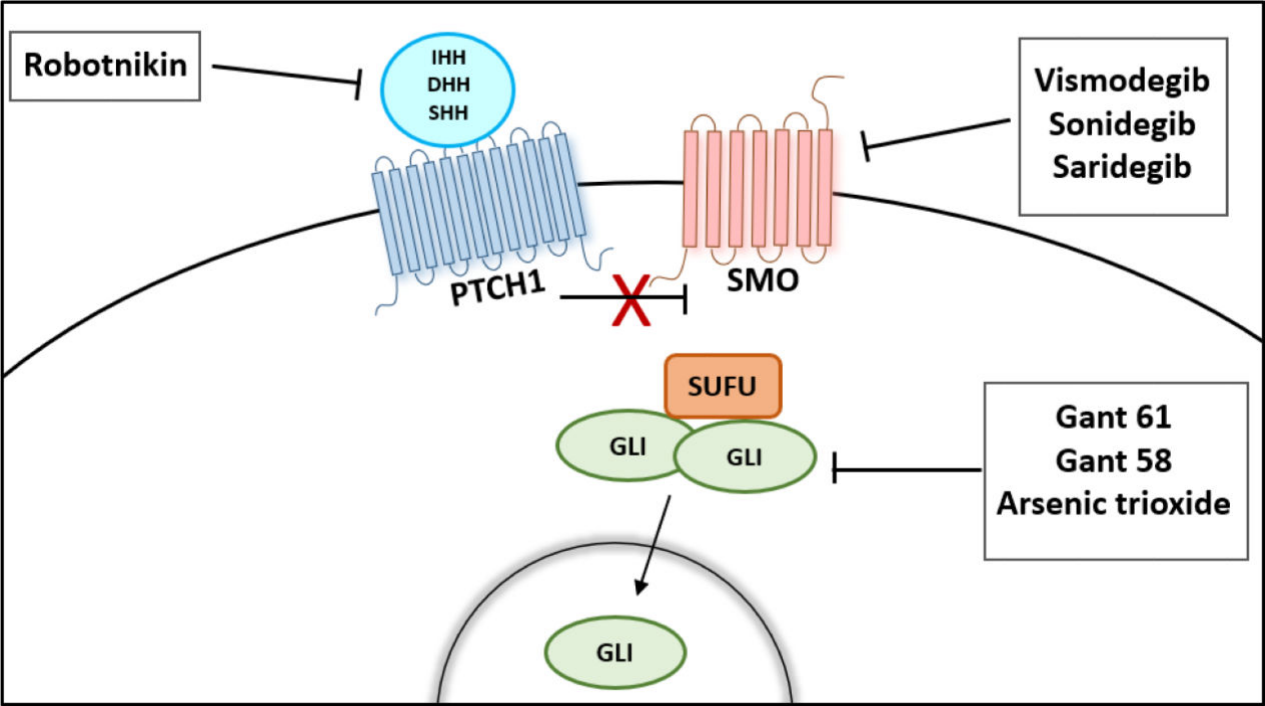
Therapeutic Options to Block the Activation of Hedgehog Signaling Pathway.

Several compounds have been identified to inhibit the HH signaling pathway and can be categorized as HH ligand inhibitors (HH neutralizing antibodies and small molecule Robotnikinin, SMO antagonists, cyclopamine and its derivatives (IPI-926 and Cyc-T), synthetic compounds such as Vismodegib (GDC0449), Sonidegib (LDE225), and GLI transcriptional inhibitors (Gant 58 and Gant 61) [58] (Figure 3).

### 4.1 Hedgehog ligand inhibitor

The only ligand inhibitor described in the literature is a small molecule called Robotnikin, which binds to an extracellular sonic HH protein. This molecule is able to bind to the ligand SHH protein, therefore blocking downstream of the signaling [59]. Currently, there are no studies reported in the literature using this drug in female cancers.

### 4.2 SMO inhibitors

SMO is the first molecule reported in the literature to target the HH pathway. Through suppression of SMO, activation of GLI transcription factors was decreased, leading to the downregulation of the HH target genes [60]. Cyclopamine is the first component described to block SMO [61, 62], its use *in vitro* and *in vivo* has shown anticancer activity. However, Cyclopamine has poor bioavailability making the clinical utility limited [63, 64]. Vismodegib (Erivedge Capsule, Genentech, Inc, USA) (GDC0449) was approved by the FDA in 2012 for the treatment of patients who are not candidates for surgery or radiation therapy, locally advanced, metastatic or recurrent basal cell carcinoma [65]. This drug is usually given until the disease progresses or until unacceptable toxicity occurs [66].

Vismodegib is a small molecule showing promising outcomes through inactivating SMO, resulting in decreased downstream target gene expression [67]. In a preclinical trial, Vismodegib, exhibited excellent potency, solubility, and metabolic stability. In addition, Phase I and II clinical trials in patients with various carcinomas have shown to have a positive response to this compound [66]. Currently, there are several clinical trials using this molecule to treat several types of female cancer (Table 1). Unfortunately, there are two known SMO mutations (D473 and E518) that can lead to resistance of vismodegib, thus decreased the ability of vismodegib to bind to SMO leading to decrease efficacy [68, 69].

Sonidegib (LDE225) was first identified in 2010 during screening biphenyl carboxamides that displayed potent antitumor activity against a medulloblastoma model [70]. In July 2015, this drug was marketed as Odomzo by Novartis. Its approval by the FDA has been used for the treatment of recurrent basal cell carcinoma in patients who are not eligible for surgery or radiotherapy. Sonidegib interacts with SMO, acting as an antagonist, preventing downstream activation of the HH pathway signaling pathway [71–73]. It has favorable blood-brain barrier penetration and high tissue penetration, making it a viable treatment for medulloblastoma [70]. Unfortunately, SMO mutations in Q476 and D473, prevent Sonidegib binding. Other mutations, including S533 and W535, confer resistance to Sonidegib [73, 74]. Currently, there are 2 ongoing clinical trials using this drug to treat breast and ovarian cancer (Table 1).

Saridegib is a potent SMO inhibitor, also known as IPI-926, a cyclopamine derivative. Studies have been shown to benefit from saridegib treatment in medulloblastoma, chondrosarcoma, and ovarian cancer [75–78]. The reduction of tumor mass in the preclinical model is explained by the decrease in the expression of GLI1 and PTCH1 [79].

### 4.3 GLI inhibitors

Gants, are the first GLI inhibitors, reported in the literature by the National Cancer Institute during GLI assay screening in HEK923 cells [80]. GLI antagonists can directly bind to GLI proteins and prevent their translocation into the nucleus. Gant 58 and Gant 61 are the most studied agents that have been used pre-clinically. They inhibit both GLI1 and GLI2 causing a significant decrease in tumor growth (80, 81). Studies have been shown that Gant 61 treatment induces cell cycle arrest by decreasing levels of the HH target such as CCND1 and increasing the expression of p21 [82, 83].

Arsenic trioxide (ATO) is an FDA-approved medication for pro-myelocytic leukemia. ATO has been found to inhibit HH signaling pathway by binding to GLI1 and GLI2 protein and prevents their binding to DNA as a transcription factor [84, 85]. ATO has been shown to increase apoptosis, reduce tumor cell growth and decrease expression of HH target genes both *in vitro* and *in vivo* [86–89].

## 5. Clinical Trials

The SHH signaling pathway has been related to several types of cancer. Clinical applications of molecules that block the SHH pathway have shown to have a significant benefit in preclinical and clinical studies to treat several types of female cancer (Table 1).

## 6. Conclusion

Although there is a remarkable growth of knowledge regarding the involvement of HH pathway in female cancer development, the precise mechanism underlying activation of HH contributing to a variety of female cancer phenotypes is mostly unknown. For instance, what are the key HH target genes related to activated canonical and non-canonical HH pathways in a variety of female cancers? What is the difference of HH regulated genes between different types of female cancer? Are there specific GLI response genes for each type of female cancer? A better understanding of these changes will lead to the development of new therapies for women with cancers.

## Figures and Tables

**Table 1:** Clinical Trials Targeting SHH Pathways in Female Cancers

Target	Drug	Cancer type	Dose	Route	Phase	Clinical trials gov. identifier	Patient recruiting status
SMO	Vismodegib	Breast cancer	400 mg	Oral	II	NCT01757327	Withdrawn
SMO	Sonidegib	Breast cancer	Unknown	Oral	I	NCT01576666	Completed
SMO	Vismodegib	Breast cancer	150 mg	Oral	I	NCT01071564	Terminated
SMO	Vismodegib	Breast cancer	Unknown	Oral	Ib	NCT03878524	Recruiting
SMO	Vismodegib	Breast cancer	150 mg	Oral	II	NCT02694224	Recruiting
SMO	Vismodegib	Breast, Ovarian, Cervical, Endometrial Cancer	Unknown	Oral	II	NCT02465060	Recruiting
SMO	Vismodegib	Ovarian	150 mg	Oral	II	NCT00959647	Completed
SMO	Vismodegib	Ovarian	150 mg	Oral	II	NCT00739661	Completed
SMO	Sonidegib	Ovarian	400, 600 and 800 mg	Oral	I	NCT01954355	Completed
SMO	Itraconazole	Ovarian	Unknown	Unknown	III	NCT03458221	Not yet recruiting
